# A Cognitive Modeling Approach to Strategy Formation in Dynamic Decision Making

**DOI:** 10.3389/fpsyg.2017.01335

**Published:** 2017-08-04

**Authors:** Sabine Prezenski, André Brechmann, Susann Wolff, Nele Russwinkel

**Affiliations:** ^1^Cognitive Modeling in Dynamic Human-Machine Systems, Department of Psychology and Ergonomics, Technical University Berlin Berlin, Germany; ^2^Special Lab Non-Invasive Brain Imaging, Leibniz Institute for Neurobiology Magdeburg, Germany

**Keywords:** dynamic decision making, category learning, ACT-R, strategy formation, reversal learning, cognitive modeling, auditory cognition

## Abstract

Decision-making is a high-level cognitive process based on cognitive processes like perception, attention, and memory. Real-life situations require series of decisions to be made, with each decision depending on previous feedback from a potentially changing environment. To gain a better understanding of the underlying processes of dynamic decision-making, we applied the method of cognitive modeling on a complex rule-based category learning task. Here, participants first needed to identify the conjunction of two rules that defined a target category and later adapt to a reversal of feedback contingencies. We developed an ACT-R model for the core aspects of this dynamic decision-making task. An important aim of our model was that it provides a general account of how such tasks are solved and, with minor changes, is applicable to other stimulus materials. The model was implemented as a mixture of an exemplar-based and a rule-based approach which incorporates perceptual-motor and metacognitive aspects as well. The model solves the categorization task by first trying out one-feature strategies and then, as a result of repeated negative feedback, switching to two-feature strategies. Overall, this model solves the task in a similar way as participants do, including generally successful initial learning as well as reversal learning after the change of feedback contingencies. Moreover, the fact that not all participants were successful in the two learning phases is also reflected in the modeling data. However, we found a larger variance and a lower overall performance of the modeling data as compared to the human data which may relate to perceptual preferences or additional knowledge and rules applied by the participants. In a next step, these aspects could be implemented in the model for a better overall fit. In view of the large interindividual differences in decision performance between participants, additional information about the underlying cognitive processes from behavioral, psychobiological and neurophysiological data may help to optimize future applications of this model such that it can be transferred to other domains of comparable dynamic decision tasks.

## Introduction

Backcountry skiers (and snowboarders) strive for the unique thrill of skiing or snowboarding down powder covered mountains, drawing the first line into freshly fallen snow. Before deciding to go down a particular mountain slope, they check the snowpack, the temperature and wind conditions to avoid setting off an avalanche. Often not a single snow characteristic is crucial but conjunctions of them can change the conditions of safe skiing. The decision to continue on a slope is re-evaluated often, depending on the feedback from the snow (e.g., collapsing snow, snow-brakes vs. nice powder snow) and previous experience.

The described scenario gives a good example of complex cognition. Complex cognition (Knauff and Wolf, 2010) investigates how different mental processes influence action planning, problem solving and decision-making. The term “mental processes in complex cognition” includes not only cognitive but also motivational aspects. Naturalistic decision-making research investigates how decisions are made “in the wild.” Real-life decisions made by people with some kind of expertise are investigated in the context of limited time, conflicting goals, dynamically changing conditions, and information sources of varying reliability.

Such complex situations involve further aspects that cannot all be covered in combination when studying complex cognition. Nevertheless, researchers should aim at describing, understanding and predicting human behavior in its complexity.

A model situated within cognitive architectures can simulate multiple parallel processes, thereby capturing multifaceted psychological phenomena and making predictions, sometimes even for complex tasks. Nevertheless, developing such models requires a stepwise procedure to distinguish different influencing factors. For our skiing example, first a model of the core decision-making process (e.g., based on category learning from snow characteristics and feedback) of a backcountry skier needs to be developed and tested. Afterwards this approach can be extended with modeling approaches of other decision influencing processes (e.g., motivation) to predict decision-making in the wild.

To come closer to the overall goal of understanding cognition as a whole, studying dynamic decision-making with cognitive architectures constitutes a step in the right direction. In dynamic decision-making, decisions are not seen as fixed but can be modified by incoming information. So, not only singular aspects of decision-making are considered, such as attentional influence, but also environmental factors that give feedback about an action or lead to major changes requiring an adaptation to new conditions.

In real-life decisions, however, our future choices and our processing of decision outcomes are influenced by feedback from the environment. This is the interactive view on decision-making, called dynamic decision-making (Gonzalez, 2017), of which the scenario presented above is an example. According to Edwards (1962), three aspects define dynamic decision-making. First, a series of actions are taken over time to achieve a certain goal. Second, the actions depend on each other. Thus, decisions are influenced by earlier actions. Third, and most difficult to investigate, changes in the environment occur as a result of these actions but also spontaneously (Edwards, 1962). According to Gonzalez (2017), dynamic decision-making is a process where decisions are motivated by goals and external events. They are dependent on previous decisions and outcomes. Thus, decisions are made based on experience and are dependent on feedback. Most of the time, these kinds of decisions are made under time constraints. Therefore, long mental elaborations are not possible. To sum up, dynamic decision-making research investigates a series of decisions which are dependent on previous decisions and are made under time constraints in a changing environment.

Another view on dynamic decision-making as a continuous cycle of mental model updating is introduced by Li and Maani (2011). They describe this process using the CER Cycle. CER stands for Conceptualization–Experimentation–Reflection. Conceptualization is obtaining an understanding of the situation and mentally simulating the outcome of potential decisions and related actions. Thus, the decision maker compares the given situation with related information in his or her mental model and integrates new information obtained from the environment to develop a set of decisions. During experimentation, the decisions and interventions devised from the decision-maker's mental model are tested in the dynamics of the real world. In the reflection phase, the outcome of the experimentation phase is reflected on, e.g., feedback is processed. If the expected outcome is achieved (e.g., positive feedback), the initial decisions are sustained. If, however, the outcome is unexpected (e.g., negative feedback) or if obtained results differ from the expected outcome, the decision maker updates his or her mental model. To do this, he or she decides for alternative actions such as searching for new sources of information for making better decisions.

These kinds of decision-making procedures have been suggested to share many processes with the procedure of category formation (Seger and Peterson, 2013). Categorization is a mental operation that groups objects based on their similar features. When new categories are formed from a given set of items without explicit instruction, the features distinguishing the different items must first be extracted. Then hypotheses about the relevant features must be formed and tested by making serial decisions.

Category learning experiments in cognitive science often require participants to establish explicit rules that identify the members of a target category. The serial categorization decisions are reinforced by feedback indicating whether a decision was correct or not. The success in such rule-based category learning experiments critically depends on working memory and executive attention (Ashby and Maddox, 2011). The fact that real world decisions critically depend on success and failure in previous trials qualifies category learning as a model for dynamic decision-making.

There are numerous advanced computational models of categorization which explain behavioral performance of subjects in various categorization tasks (e.g., Nosofsky, 1984; Anderson, 1991; Ashby, 1992; Kruschke, 1992; Nosofsky et al., 1994; Erickson and Kruschke, 1998; Love et al., 2004; Sanborn et al., 2010). These competing models differ in their theoretical assumptions (Lewandowsky et al., 2012) and there is currently no consensus on how different models can be compared and tested against each other (Wills and Pothos, 2012).

Another requirement for dynamic decision-making is the occurrence of changes in the environment. A well-known categorization task using such changes is implemented in the Wisconsin Card Sorting Test (WCST; Berg, 1948). In this test, participants must first select a one-feature rule (color, shape, number of symbols) and are then required to switch to a different one-feature rule. This task tests for the ability to display behavioral flexibility. Another experimental approach to test for behavioral flexibility in humans and animals is reversal learning (e.g., Clark et al., 2004; Jarvers et al., 2016). Here, subjects need to adapt their choice behavior according to reversed reinforcement contingencies.

Thus, category learning experiments with changing rules can serve as suitable paradigms to study dynamic decision-making in the laboratory, albeit with limited complexity as compared to real world scenarios.

The majority of rule-based category learning experiments are simple and only use one relevant stimulus feature specification (e.g., a certain color of the item) as categorization basis. In principle, however, such a restriction is not required and rule-based category learning experiments can become more complex by using conjunction rules. These can still be easily described verbally (e.g., respond A if the stimulus is small on dimension x and small on dimension y). It has been shown that conjunction rules can be learned (e.g., Salatas and Bourne, 1974) but are much less salient and are not routinely applied (Ashby et al., 1998).

In the following, the main points mentioned above are integrated in our backcountry skiing example: Since feedback from the environment plays a central role in building a correct mental model, feedback in the form of great powder snow indicates that the current strategy is correct. By contrast, negative feedback, for example breaking snow, indicates that one should change the strategy, perhaps search for different feature specifications or even for a different combination of features that might promise a better outcome for skiing. Furthermore, sudden changes of environmental conditions can result in a change of which feature combinations are indicative of a positive outcome. In our example, a change could be a different hill side with more exposure to the sun or a rise in temperature, requiring that other feature combinations should be taken as an indication for a safe descent. There are a lot of possibilities which features and feature combinations could indicate safe or unsafe conditions, making such a task complex.

Thus, to study dynamic decision-making in a category learning experiment requires a task with the above-mentioned characteristics (successive decisions with feedback, multiple feature stimuli, and switching of category assignments). To determine how humans learn feature affiliation in a dynamic environment and to investigate how strategies with rising complexity emerge, a modeling approach addressing these aspects first needs to be developed. If this model is useful and plausible it should match average behavioral data. This is an important milestone toward a more precise model which in turn should predict more detailed empirical data (e.g., individual behavioral or neural data). If this step is achieved, then models can be used as decision aiding systems at an individual level.

In this paper, we use the behavioral data of an experiment, described in the following, to develop an initial cognitive model as described above. In the experiment, a large variety of multi-feature auditory stimuli were presented to participants in multiple trials. The participants were then required to learn by trial and error which combinations of feature specifications predict a positive or negative outcome. Since perceptual learning of stimulus features is not the focus of our research, we used salient and easy-to-recognize auditory features. To meet all of the above-mentioned criteria for dynamic decision-making, we further introduced a spontaneous change in the environment such that previous decisions on feature combinations suddenly needed to be re-evaluated to obtain positive feedback.

In particular, we would like to demonstrate how different aspects that influence dynamic decision-making can be addressed through a combination of existing and validated cognitive mechanisms within an architecture. These are: learning to distinguish positive and negative feature combinations depending on feedback; successive testing of simple one-feature rules first and switching to more complex two-feature rules later, and using metacognition to re-evaluate feature combinations following environment changes. Other modeling approaches are also able to replicate such data, what distinguishes our approach is that it has a theory grounded interpretation of plausible cognitive mechanisms.

### Why use cognitive modeling?

The method of cognitive modeling forces precision of vague theories. For scientific theories to be precise, these verbal theories should be formally modeled (Dimov et al., 2013). Thus, theories should be constrained by describable processes and scientifically established mechanisms. As Simon and Newell (1971) claim, “the programmability of the theories is a guarantee of their operationality and iron-clad insurance against admitting magical entities in the head” (p. 148).

Cognitive models can make predictions of how multiple aspects or variables interact and produce behavior observed in empirical studies. In real-life situations, multiple influences produce behavior. Cognitive models are helpful to understand which interrelated cognitive processes lead to the observed behavioral outcome. Cognitive models can perform the same task as human participants by simulating multiple ongoing cognitive processes. Thereby, models can provide insight into tasks that are too complex to be analyzed by controlled experiments. Nevertheless, studying such a task with participants is mandatory to compare the outcomes of models and participants. However, understanding the process leading to an outcome is more important than perfectly fitting a model to a given set of experimental results. Our goal in this regard is to understand the processes underlying human decision-making, not least to aid humans in becoming better at decision-making (Wolff and Brechmann, 2015).

Predictions made by cognitive models cannot only be compared to average outcome data (such as reaction times, or percentage of correct decisions) but also to process data. Process data represent patterns of information search, e.g., neural data. In this regard, cognitive models can be informed by EEG and fMRI data to achieve an empirical validation of such processes (Forstmann et al., 2011; Borst and Anderson, 2015).

The development of neurobiologically plausible models is specifically the focus of reinforcement learning (e.g., Sutton and Barto, 1998). The aim of such computational models is to better understand the mechanisms involved on the neural network level as studied using invasive electrophysiological measures in different brain regions in animals (e.g., sensory and motor cortex, basal ganglia, and prefrontal cortex). Such neural network models have recently been applied to learning tasks requiring flexible behavior (e.g., contingency reversal tasks). The reader is referred to a recent paper by Jarvers et al. (2016) that gives an overview of the literature on reversal learning and describes a recurrent neural network model for an auditory category learning task such as the one applied in the current paper. This probabilistic learning model resulted in a good fit to the empirical learning behavior, but does not interpret the cognitive processes that lead to this behavior. It postulates an unspecified metacognitive mechanism that controls the selection of the appropriate strategy. This is where the strength of our approach comes into play; It is specific about the metacognitive mechanisms that drive behavior in such tasks. An example would be processes which assure that after a number of negative results, a change in strategy will be initiated.

To summarize, cognitive modeling is a falsifiable methodology for the study of cognition. In scientific practice, this implies that precise hypotheses are implemented in executable cognitive models. The output of these models (process as well as product) is then compared to empirical data. Fit-Indices such as *r*^2^ and RSME as well as qualitative trends provide information on the predictive power of the cognitive models.

More specific, the central goals of cognitive modeling are to (a) describe (b) predict, and (c) prescribe human behavior (Marewski and Link, 2014). A model that *describes* behavior can replicate the behavior of human participants. If the model, however, reproduces the exact behavior found in the human data, this is an indication of overfitting. In this case, the model has parameters that also fit the noise found in the empirical data. To address such issues of over specified models, it is important to test the model on a new data set and thereby evaluate how well it can *predict* novel data. *Prescribe* means that the model should be a generalizable model so that it can predict behavior in different situations. Moreover, robust models are preferable this implies that the output of the model is not easily influenced by specific parameter settings.

The term cognitive model includes all kinds of models of cognition—from very specific, isolated cognitive aspects only applicable in specific situations to more comprehensive and generalizable ones. The latter candidates are cognitive architectures that consider cognition as a whole. They aim at explaining not only human behavior but also the underlying structures and mechanisms. Cognitive models written on the basis of cognitive architectures therefore generally do not focus on singular cognitive processes, such as some specific learning process. By contrast, interaction of different cognitive processes and the context of cognitive processes are modeled together. Modeling the relations between different subsystems is especially relevant for applied research questions. The structures and mechanism for this are provided by the cognitive architecture and should be psychologically and neurally plausible (Thomson et al., 2015).

The most commonly used cognitive architectures, such as ACT-R, predict processes at a fine-grain level in the range of 50 ms. These processes can be implemented computationally. However, they are embedded in cognitive theories—this is what distinguishes cognitive models built with cognitive architectures from mathematical models such as neural networks. The latter models formally explain behavior in terms of computational processes. Thus, their explanation of behavior can be seen in terms of computational processes but do not aim at cognitive interpretations (Bowers and Davis, 2012).

### The cognitive architecture ACT-R

The cognitive architecture ACT-R (Adaptive Control of Thought—Rational) has been used to successfully model different dynamic decision-making tasks and is a very useful architecture for modeling learning (Anderson, 2007; Gonzalez, 2017). In the following, a technical overview of the main structures and mechanisms that govern cognitive models in ACT-R is given. We will focus only on those aspects that are important to understand our modeling approach. For a more detailed insight into ACT-R, we recommend exploring the ACT-R website^1^.

ACT-R's main goal is to model cognition as a whole using different modules that interact with each other to simulate cognitive processes. These modules communicate via interfaces called buffers. ACT-R is a hybrid architecture, thus symbolic and subsymbolic mechanisms are implemented in the modules of ACT-R.

Our model uses the motor, the declarative, the imaginal, the goal, the aural^2^, and the procedural module. The motor module represents the motor output of ACT-R. The declarative module is the long-term memory of ACT-R in which all information units (chunks) are stored and retrieved. The imaginal module is the working memory of ACT-R in which the current problem state (an intermediate representation important for performing a task) is held and modified. Thus, the imaginal module plays an important role for learning. The goal module holds the control states. These are the subgoals that have to be achieved for the major goal. The aural module is the perceptual module for hearing. The procedural module plays a central role in ACT-R. It is the interface of the other processing units, since it selects production rules (see below) based on the current state of the modules.

Writing a model requires the modeler to specify the symbolic parts of ACT-R. These are (a) the production rules, and (b) the chunks. Chunks are the smallest units of information. All information in ACT-R is stored in chunks. Production rules (e.g., productions) consist of a condition and an action part. Productions are selected sequentially, and only one production can be selected at a given time. A production can only be selected if the condition part of the production matches the state of the modules. Then, the action part modifies the chunks in the modules. If more than one production matches the state of the modules then a subsymbolic production selection process chooses which of the matching productions is selected.

A further subsymbolic process in ACT-R is the activation of a chunk. It determines if a chunk can be retrieved from memory and how long this retrieval takes. The past usefulness of a chunk (base-level activation), the chunk's relevance in the current context (associative activation) and a noise parameter sum up to the chunk's activation value. Modifying the subsymbolic mechanisms of ACT-R is also part of the modeling procedure. This can be done using specific parameters—however, most parameters have default values derived from previous studies (Wong et al., 2010) which should be used.

### How can decision-making and category learning be modeled in ACT-R?

Many different styles for writing models in ACT-R exist (Taatgen et al., 2006). The following modeling approaches have been used for decision-making: (a) strategy or rule-based, (b) exemplar or instance-based, and (c) approaches that mix strategies and exemplars. These approaches will be compared to motivate our chosen modeling approach.

In *strategy or rule-based models*, different problem solving strategies are implemented with different production rules and successful strategies are rewarded. Rule-based theories in category learning postulate that the categorizer must identify the category of an object by testing it against different rules. So, to find a solution for a problem, strategies in the form of rules are used.

*Exemplar or instance-based models* rely on previous experience stored in declarative memory to solve decision-making problems. The content and structure of the exemplars depend on individual framing. It is not a complete representation of the event, but represents the feature specifications the problem solver is focused on, together with experienced feedback. Exemplar theories of category learning postulate that category instances are remembered. To decide if an instance belongs to a category, a new instance is compared to an existing instance. Instance-Based Learning (IBL) builds upon instances in the context of dynamic decision processes and involves learning mechanisms such as recognition-based retrieval. The retrieval of instances depends upon the similarity between the current situation and instances stored in memory. In IBL situations, outcome observations are stored in chunks and retrieved from memory to make decisions. The subsymbolic activation of the retrieved instances determines which instances are likely to be retrieved in a given situation. Instance Based Learning requires some amount of previous learning of relevant instances. Then, decision makers are able to retrieve and generalize from these instances (Gonzalez et al., 2003).

*Mixed approach models* use both rules and instances to solve decision-making problems.

Several authors implemented the described approaches in category learning and decision-making environments. In a strategy-based ACT-R model, Orendain and Wood (2012) implemented different strategies for complex problem solving in a microworld^3^ game called “Firechief.” Their model mirrored the behavior of participants in the game. Moreover, different training conditions and resulting behavior of the participants could be modeled. The model performed more or less flexibly, just as the participants, according to different training conditions. This demonstrates that success in strategy learning depends on the succession of stimuli in training conditions. Peebles and Banks (2010) used a strategy-based model of the dynamic-stocks and flows task (DSF). In this task, water level must be held constant but the inflow and outflow of the water changes at varying rates. An ACT-R model of strategies for accomplishing this task was implemented in form of production rules. The model replicated the given data accurately, but was less successful in predicting new data. The authors proposed that by simply extending the model so it contains more strategies and hypotheses, it would be able to predict such new data as well. Thus, specifying adequate rules is crucial for rule-based models.

Gonzalez et al. (2009) compared the performance of two ACT-R models, an instance-based model and a strategy-based model, in a RADAR task. In this task, participants and the model had to visually discriminate moving targets (aircrafts) among moving distractors and then eliminate the targets. Both models achieved about the same overall fit to the participants' data, but IBL performed better in a transfer task.

Lebiere et al. (1998) tested two exemplar models that captured learning during a complex problem-solving task, called the sugar factory (Berry and Broadbent, 1988). The sugar factory task investigates how subjects learn to operate complex systems with an underlying unknown dynamic behavior. The task requires subjects to produce a specific amount of sugar products. Thus, in each trial the workforce needs to be adjusted accordingly. The two exemplar models produced adequate learning behavior similar to that of the subjects. In a subsequent study, Fum and Stocco (2003) investigated how well these original models could predict participants' behavior in case of a much lower target amount of the sugar product than in the original experiment. Furthermore, they investigated if the models could reproduce behavior in case of switching from a high product target amount to a low product target amount and vice versa during the experiment. The performance of the participants increased significantly in the first case. The original IBL models were not able to capture this behavior. The authors therefore developed a rule based model that captured the subjects, switching behavior.

Rutledge-Taylor et al. (2012) compared a rule-based and an exemplar-based model for an intelligence categorization task where learned characteristics had to be studied and assigned. Both models performed equally well in predicting the participants' data. No model was superior to the other.

In a different categorization study, Anderson and Betz (2001) studied three category-learning tasks with three different ACT-R models, an exemplar-based model, a rule-based model and a mixed model. The mixed model fitted best, reproducing learning and latency effects found in the empirical data.

In summary, there is no clear evidence that one or the other modeling approach is superior. In their paper, Anderson and Betz (2001) state that the mixed approach is probably the closest to how humans categorize, because the assumption that categorization is either exclusively exemplar-based or exclusively rule-based is probably too close-minded. Furthermore, stimulus succession and adequate rule specification are important for dynamic decision-making and category learning tasks.

In addition, models of complex tasks should incorporate metacognitive processes such as reflecting and evaluating the progress of the selected approach (Roll et al., 2004; Reitter, 2010; Anderson and Fincham, 2014). Reitter's (2010) model of the dynamic stocks and flow tasks investigated how subjects manage competing task strategies. The subject-to-subject analysis of the empirical data showed that participants exhibited sudden marked changes in behavior. Learning mechanisms which are purely subsymbolic cannot explain such behavior, because changes in model behavior would take too long. Furthermore, the strategies of the participants seemed to vary with the complexity of the water flow. Thus, a model of this task must address switches in strategy and not only gradual learning. Reitter (2010) assumes that humans' solutions to real-world problems emerge from a combination of general mechanisms (core learning mechanisms) and decision-making strategies common to many cognitive modeling tasks. His model implements several strategies to deal with the basic control task as well as a mechanism to rank and select those strategies according to their appropriateness in a given situation. This represents the metacognitive aspect of his model.

### Our aim

Our aim is to develop an ACT-R modeling approach for dynamic decision-making in a category learning task. A suitable task for such a modeling approach needs to fulfill several requirements. First, it should use complex multi-feature stimuli for the model to build categories from combined features. Second, the task needs to provide feedback, thereby allowing the model to learn. Third, changes in the environment should occur during the task forcing the model to act on them by refining once learned category assemblies.

To model performance in such a task, the modeling approach will need to incorporate mechanisms for strategy learning and strategy switching. It should precisely specify how hypotheses about category learning can be implemented with ACT-R. A mixed modeling approach of rules and exemplars should be used since previous work indicates that such models are most suitable for dynamic decision-making tasks. Furthermore, since switches in category assignments as well as monitoring of the learning progress need to be addressed, metacognitive aspects should be incorporated in the modeling approach.

Our modeling approach should provide information on the actual cognitive processes underlying human dynamic decision-making. Hence, it should be able to predict human behavior and show roughly the same performance effects that can be found in empirical data reflecting decision-making, e.g., response rates. Even more importantly, we aim at developing a general model of dynamic decision-making. For the model to be general (e.g., not fit exclusively to one specific experimental setting or dataset), it needs to be simple. Thus, only few assumptions should be used and unnecessary ones avoided. As a result, the modeling approach should be capable to predict behavior with other stimulus materials and be transferable to other similar tasks.

To summarize the scope of this article, our proposed modeling approach aims to depict the core processes of human decision-making, such as incorporating feedback, strategy updating, and metacognition. Building a model with a cognitive architecture ensures that evaluated cognitive processes are used. The quest is to see whether these cognitive aspects including the processes of the architecture can produce empirical learning behavior:

First, performance improvement through feedback should be included in the model. In the case of feature learning and strategy updating, improvements in one's strategy are only considered in the case of negative feedback (Li and Maani, 2011). If feedback signals a positive decision, people consider their chosen strategy for later use. Thus, people update their mental model during dynamic decision-making only if they receive negative feedback (Li and Maani, 2011). For our feature learning model, this implies that once a successful strategy has been chosen over alternatives, revisions to this strategy will require negative feedback on that strategy rather than positive experience with others, as these are no longer explored.

Second, the model should include transitions from simple to complex strategies. Findings suggest that people initially use simple solutions and then switch to more complex ones (Johansen and Palmeri, 2002). The modeling approach under discussion should be constructed in a similar fashion. In the beginning, it should follow simple one-feature categorization strategies and later switch to more complex two-feature strategies.

Third, the model needs to use metacognitive mechanisms. For example, it needs specifications for which conditions switching from a single-feature strategy to a multi-feature strategy is required. The metacognitive aspects should furthermore reflect previous learning successes. Thus, keeping track of which approaches were helpful and which were not, or of how often a strategy has been successful in the past, should be implemented in the model. Moreover, such mechanisms should ensure that if a strategy was successful in the past and fails for the first time, it is not discarded directly, but tested again. Furthermore, metacognitive mechanisms should not only address the issue of switching from single-feature to multi-feature strategies but also incorporate responses to changes in the environment.

## Materials and methods

In the following, an experiment of dynamic decision-making and our model performing the same task are presented. The model includes mechanisms to integrate feedback, to switch from simple to complex strategies and to address metacognition. The model was built after the experimental data were obtained.

This section is subdivided in the following manner: First, the participant sample, setup and stimuli of the empirical experiment are described. Then, the modeling approach is explained in detail. Afterwards, the model setup and stimuli are presented. Finally, the analytical methods to evaluate the fit between the model and the empirical results are outlined.

### Experiment participants

55 subjects participated in the experiment that took place inside a 3 Tesla MR scanner^4^ (27 female, 28 male, age range between 21 and 30 years, all right handed, with normal hearing). All subjects gave written informed consent to the study, which was approved by the ethics committee of the University of Magdeburg, Germany.

### Experimental stimuli

A set of frequency-modulated different tones served as stimuli for the categorization task. The tones differed in duration (short, 400 ms, vs. long, 800 ms), direction of frequency modulation (rising vs. falling), intensity (low intensity, 76–81 dB, vs. high intensity, 86–91 dB), frequency range (five low frequencies, 500–831 Hz, vs. five high frequencies, 1630–2639 Hz), and speed of modulation (slow, 0.25 octaves/s, vs. fast, 0.5 octaves/s), resulting in 2 × 2 × 2 × 10 × 2 (160) different tones. The task relevant stimulus properties were the direction of frequency modulation and sound duration, resulting in four tone categories: short/rising, short/falling, long/rising, and long/falling. For each participant, one of these categories constituted the target sounds (25%), while the other three categories served as non-targets (75%).

As feedback stimuli, we used naturally spoken utterances (e.g., ja, “yes”; nein, “no”) as well as one time-out utterance (zu spät, “too late”) taken from the evaluated prosodic corpus MOTI (Wolff and Brechmann, 2012, 2015).

### Experimental paradigm

The experiment lasted about 33 min in which a large variety of frequency-modulated tones (see Section Experimental Stimuli above) were presented in 240 trials in pseudo-randomized order and with a jittered inter-trial interval of 6, 8, or 10 s. The participants were instructed to indicate via button-press whether they considered the tone in each trial to be a target (right index finger) or a non-target (right middle finger). They were not informed about the target category but had to learn by trial and error. Correct responses were followed by positive feedback, incorrect responses by negative feedback. If participants failed to respond within 2 s following the onset of the tone, the time-out feedback was presented.

After 120 trials, a break of 20 s was introduced. From the next trial on the contingencies were reversed such that the target stimulus required a push of the right instead of the left button. The participants were informed in advance about a resting period after finishing the first half of the experiment but they were not told about the contingency reversal.

### Model in detail

In the following, the model is presented in detail. First, a description of the main declarative representations (chunks) is provided. They reflect strategy representations and metacognitive processes. This is followed by a description of how the model runs through a trial. Finally, the rules that govern strategy learning are summarized.

#### Chunks and production rules used in the model

**Figure 1:**
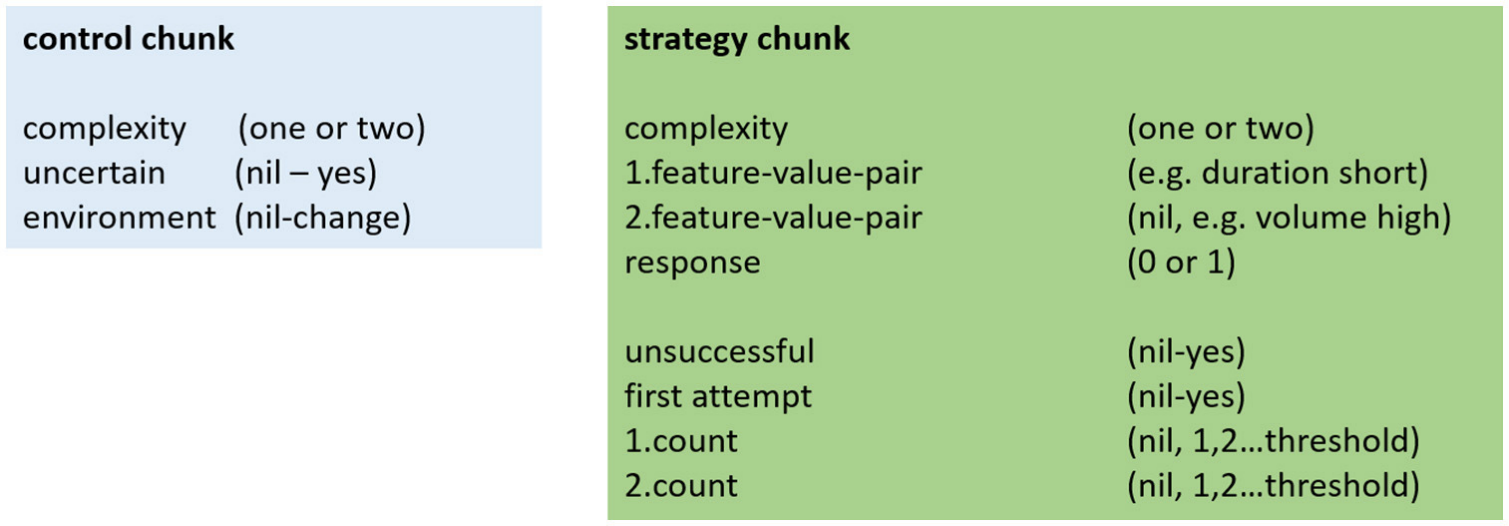
Schematic build-up of the structure of the control and the strategy chunk. Nil indicates that the variable has no value.

The chunks implemented in the model are shown in Figure 1. “Strategy chunks” hold the strategies in form of examples of feature-value pairs and responses. They are stored in and retrieved from long-term memory (declarative module). The current strategy is held in working memory (imaginal module). Strategy chunks contain the following information about the strategy: which feature(s) and what corresponding value(s) are relevant (e.g., the sound is loud or the sound is loud and its frequency range is high), what the proposed response is (categorization, 1 or 0), and the degree of complexity of the strategy (e.g., one or two-feature strategy). Furthermore, an evaluation mechanism is part of this chunk. This includes noting if a strategy was unsuccessful and keeping track of how often a strategy was successful. This tracking mechanism notices if the first attempt to use this strategy is successful. It then counts the number of successful strategy uses; this explicit count is continued until a certain value is reached. We implemented such a threshold count mechanism to reflect the subjective feeling that a strategy was often useful. We implemented different threshold values for the model. We also differentiated between the threshold for one-feature strategies (first count) and for two-feature strategies (second count). The tracking mechanism can be seen as a metacognitive aspect of our model. Other metacognitive aspects are implemented in the “control chunk” which is kept in the goal buffer of the model. These metacognitive aspects include: first, the level of feature-complexity of the strategy, i.e., if the model attempts to solve the task with a one-feature or with a two-feature strategy; second, whether or not a long-time successful strategy caused an error, this signifies the model's uncertainty about the accuracy of the current strategy; third, whether changes in the environment occurred that require to renew the search for an adequate strategy.

#### Trial structure

**Figure 2:**
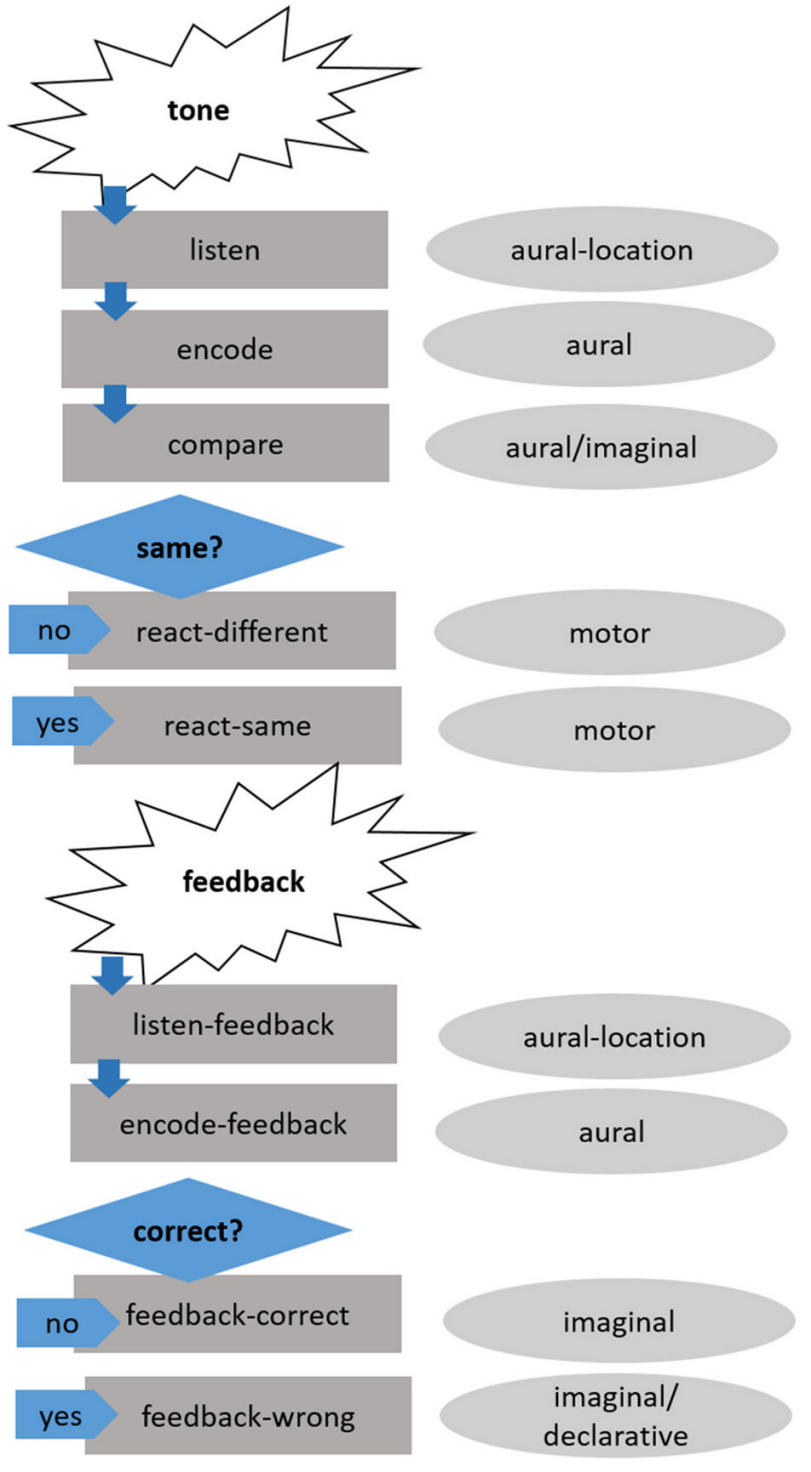
Schematic overview of how the model runs through a trial. The dark-gray boxes on the left represent the production rules, the light-gray ovals on the right the main buffers involved.

Production rules govern how the model runs through the task. The flow of the model via its production rules is illustrated in Figure 2. The following section describes how the model runs through a trial, the specific production rules are noted in parentheses.

A tone is presented to the model and enters the aural-location buffer *(listen)*. After the tone has finished, it is encoded in the aural buffer *(encode)*. Thus, a chunk with all audio information necessary (duration, direction of pitch change, intensity, and frequency range—see Section Modeling Paradigm and Stimuli below) is in the aural buffer and all four characteristics of the tone are accessible to the model. The audio chunk in the aural buffer is then compared to the strategy chunk held in the imaginal buffer *(compare)*. If the specific features (e.g., intensity is high) of the strategy chunks are the same as in the audio chunk, the response is according to the strategy proposed by the model *(react-same)*, if not, the opposite response is chosen *(react-different)*. The presented feedback is listened to and held in the aural-location buffer *(listen-feedback)* and then encoded in the aural buffer *(encode-feedback)*. If the feedback is positive, the current strategy is kept in the imaginal buffer and the count-slot is updated *(feedback-correct)*. If the feedback is negative, the strategy is updated depending on previous experiences *(feedback-wrong)*. Thus, a different strategy chunk is retrieved from declarative memory and copied to the imaginal buffer.

#### Finding an adequate strategy

**Figure 3:**
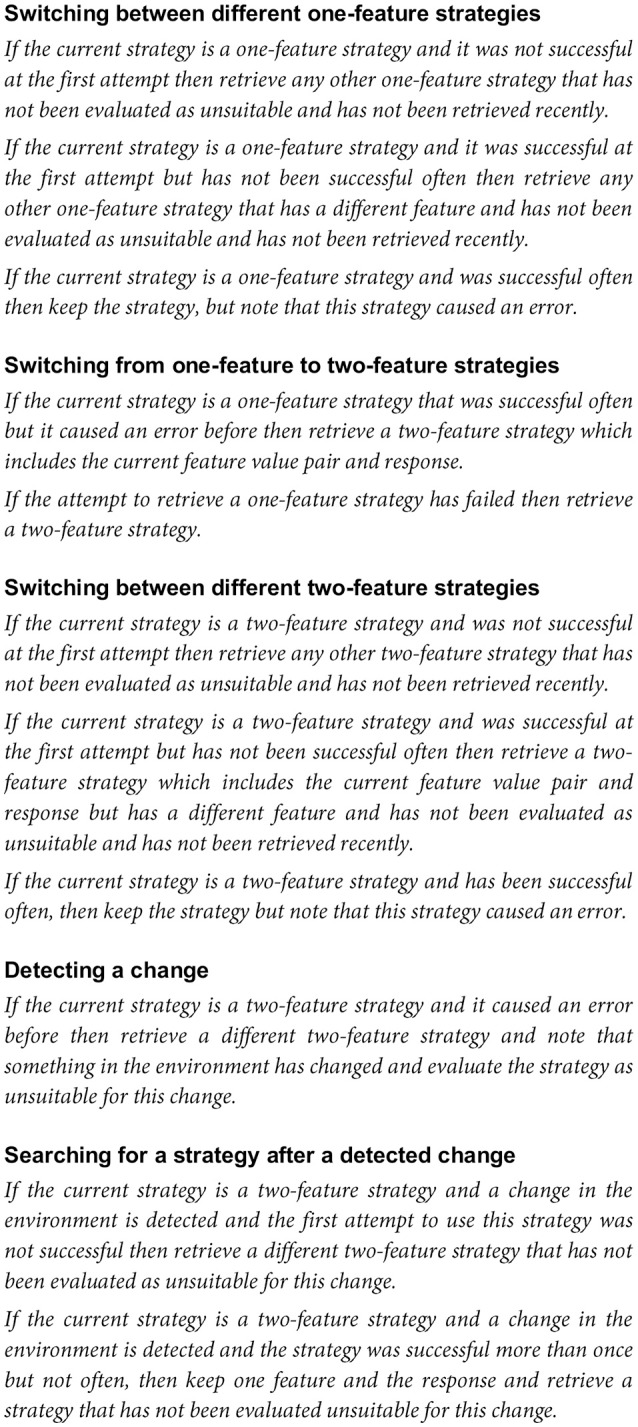
Rules governing when and to what degree the strategies are changed after negative feedback is received.

All possible strategies are already available in the model's long-term memory. The currently pursued strategy is maintained in working memory and evaluated regarding the feedback. For positive feedback, the strategy is retained and it is counted how often it is successful. If feedback is negative, the strategy is usually altered. The following subsection is a summary of how strategy updating is implemented. For more information see Figure 3.

The model always begins with a one-feature strategy (which strategy it begins with is random) and then switches to another one-feature strategy. The nature of the switch depends on how often a particular strategy was successful. When the model searches for different one-feature strategies, it retrieves only strategies which were not used recently. In case of immediate failure of a one-feature strategy, a different response is used for the feature-value pair. In other cases, the feature-value pair is changed, but the response is retained. If a one-feature strategy has been successful often and then fails once, the strategy is not directly exchanged, but re-evaluated. However, it is also noted that the strategy has caused an error. Two possibilities explain why switches from a one-feature to a two-feature strategy occur: Such a switch can happen either because no one-feature strategy that was not negatively evaluated can be retrieved or because an often successful one-feature strategy failed repeatedly. Switches within the two-feature strategy are modeled the following way: If a two-feature strategy was unsuccessful at the first attempt, any other two-feature strategy is used (which one exactly is random). If a two-feature strategy was initially successful and then fails, then a new strategy which retains one of the feature-value pairs and the response will be selected. This strategy only differs in the other feature-value pair. When the environment changes, a previously often successful two-feature strategy (and also a one-feature strategy) will fail. Then a retrieval of another two-feature strategy is attempted. If at the time the environment changes, the model has not found a successful two-feature strategy, it will continue looking for a useful two-feature strategy, and thus not notice the change.

### Modeling paradigm and stimuli

The following section briefly describes how the experiment was implemented for the model. This includes a short overview of how the stimulus presentation was modified for the model.

The task of the participants was implemented for the model in ACT-R 7.3 with some minor modifications. The same four pseudo-randomizations used for the participants were also used for the model. Thus, 25% of the stimuli were target stimuli. A trial began with a tone, which lasted for 400 ms. To model the two stimulus durations, we used two different features in the new-other-sound command. As soon as the model responded via button press, auditory feedback was presented. Overall, a trial lasted for a randomized period of 6, 8, or 10 s, similar to the original experiment. There was no break for the model after 120 trials, but the targets switched after 120 trials, too.

Instead of employing all 160 different tones, sixteen different tones were presented to the model. Each of the tones is a composition of four characteristics of the four binary features: duration (long vs. short), direction of frequency modulation (rising vs. falling), intensity (low intensity, vs. high intensity), and frequency range (low vs. high). Only binary features were used for the model because the perceptual difference between the two classes of each selected feature was high, except for speed of modulation, which was therefore not implemented in the model. For the participants, more feature variations were used to ensure categorical decisions and to prevent them from memorizing individual tone-feedback pairs. This is not an issue for the model, since no mechanism allowing such memorizing was implemented. As for the participants, auditory feedback was presented to the model.

The modeling approach is a mixed modeling approach, the strategies are encoded as instances, but which instance is retrieved is mainly governed by rules.

To test if the model is a generalizable model, different variations were implemented. The learning curves found in the empirical data should still be found under different plausible parameter settings. However, specific parameter settings should influence the predictive quality of the model. The approach typically chosen by cognitive modelers is to search for specific parameter settings that result in an optimal fit and then report this fit. The objective behind such an approach is to show that the model resembles the ongoing cognitive processes in humans. We have chosen a different approach. Our objective is to show that our modeling approach can map the general behavior such as learning and reversal learning as well as variance found in the data. By varying parameter settings, we want to optimize the fit of the model and examine the robustness of the model mechanisms to parameter variations.

Regarding the choice of varying parameters, we use an extended parameter term which includes not only subsymbolic ACT-R parameters (which are typically regarded as parameters) but also certain (production) rules (Stewart and West, 2010). In the case of this model, productions that control the tracking mechanism of successful strategies are varied. The tracking mechanism keeps track of how often a strategy is successful. However, the model does not increase the count throughout the entire experiment. After it reaches a threshold, a successful strategy is marked as “successful often.” Thereafter, it is not discharged directly in case of negative feedback but instead reevaluated. So, to answer the question what the most suitable values for the threshold of the first and second count are, these values were varied. Another implemented model assumption is that this threshold is different for single-feature vs. two-feature strategies. We assumed that the threshold for two-feature strategies should be double the value for one-feature strategies, as if the model was counting for each feature separately. The first count was varied for three, four and five and the second count for six, eight, and ten.

Besides the parameters that control the tracking mechanism, we also investigated a parameter-controlled memory mechanism. The latter controls for how long the model can remember if it had already used a previous strategy. This is the *declarative-finst-span*^5^ parameter of ACT-R. We assumed that participants remember which strategy they previously used for around 10 trials back. We therefore tested two different values (80 and 100 s) for this parameter, determining whether the model can remember if this chunk has been retrieved in the last 80 (or 100) s. The combination of the declarative-finst-span (80, 100), three values for the first count (3, 4, 5) and three values for the second count (6, 8, 10) resulted in 18 modeling versions (see Table 1).

**Table 1:** Resulting modeling versions from combining the different parameter settings for the first and second count and the declarative-finst-span.

		**First count 3**	**First count 4**	**First count 5**
:declarative-finst-span 80	Second count 6	3_06 _080	4_06 _080	5_06 _080
	Second count 8	3_08 _080	4_08 _080	5_08 _080
	Second count 10	3_10 _080	4_10 _080	5_10 _080
:declarative-finst-span 100	Second count 6	3_06 _100	4_06 _100	5_06 _100
	Second count 8	3_08 _100	4_08 _100	5_08 _100
	Second count 10	3_10 _100	4_10 _100	5_10 _100

### Analyses

Each of the models was run 160 times, 40 times for each pseudo-randomized order, using ACT-R 7.3. The data were preprocessed with custom Lisp files and then analyzed with Microsoft Excel.

The model data and the empirical data were divided into 12 blocks, with 20 trials per block. The average proportion of correct responses and the standard deviation per block was computed for the experiment as well as for each of the 18 models.

One aim of this study was to predict average learning curves of the participants. Thus, the proportion of correct responses of the participants was compared to the proportion of correct responses of each of the models. Visual graphs comparing the modeled to the empirical data were analyzed with regard to increases and decreases in correct responses.

As an indication of relative fit, the correlation coefficient (*r*) and the determination coefficient (*r*^2^) were computed. They represent how well trends in the empirical data are captured by the model.

As an indication of absolute fit, the root-mean-square error (RMSE) was calculated. RMSE represents how accurately the model predicts the empirical data. RMSE is interpreted as the standard deviation of the variance of the empirical data that is not explained by the model.

To compare the participant-based variance found in the empirical data with the variance produced by the 160 individual model runs, a Levene's test (a robust test for testing the equality of variances) was calculated for each block of the experiment.

## Results

In the following sections, the empirical data, the modeled learning curves, and the results regarding the general fit of the different model versions to the data are presented.

### Empirical learning curves

**Figure 4:**
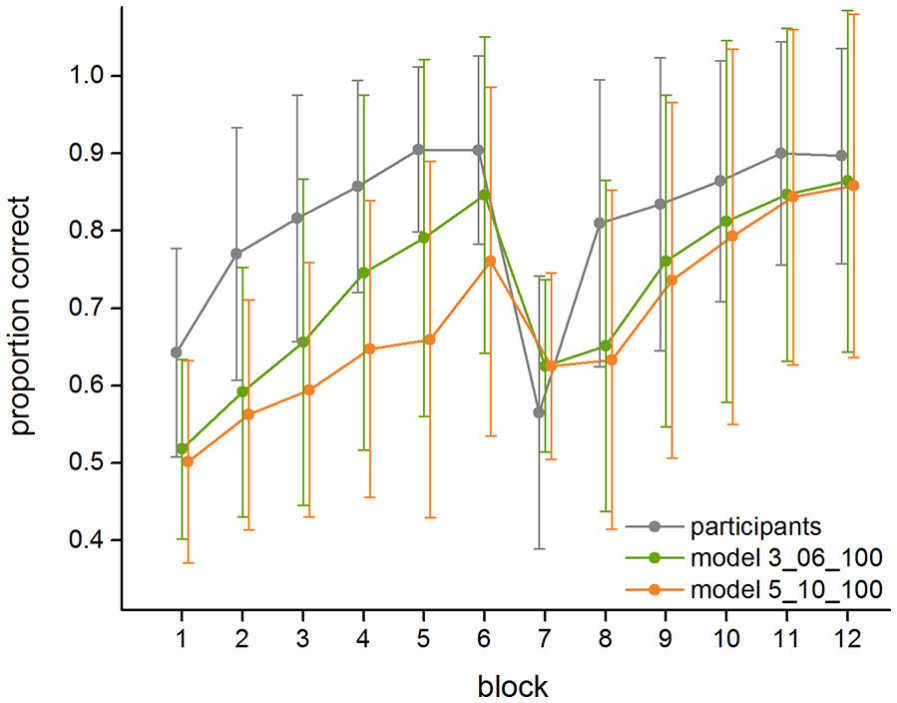
Average performance and standard deviations of the human participants, the best fitting model (3_06_100), and the worst fitting model (5_10_100) in the 12 blocks of the experiment.

The descriptive analysis of the empirical data (see Figure 4 and Table 2) shows that on average, in the first block the participants respond correctly in 64.3% (±13.5%) of the trials. The response rate of the participants increases until the sixth block to 90.4% (±12.2%) of correct trials. In the seventh block, the block in which targets and non-targets switch, it drops to 56.5% (±17.7%) of correct trials. It then increases again and reaches 81.0% (±18.5%) of correct trials in the eighth block and 89.7% (±13.9%) of correct trials in the last block. Across all 12 blocks, the standard deviation of the empirical data ranges from 10.7% minimum to 18.9% maximum, with an average standard deviation of 15.1%. The standard deviation of the participants derives from the fact that different participants showed different learning curves, and not all participants reported to have found the correct strategy in a post interview. Correspondingly, eleven participants (20.0%) showed a performance below 85% by the end of the first part of the experiment (Block 6), and 12 participants (21.8%) stayed below 85% correct responses at the end of the second part (Block 12).

**Table 2:** Average proportion of correct responses and standard deviations (in %) of the participants and the 18 versions of the model in the 12 blocks of the experiment.

	**1**	**2**	**3**	**4**	**5**	**6**	**7**	**8**	**9**	**10**	**11**	**12**
Participants	64.3 ± 13.5	77.0 ± 16.4	81.6 ± 15.9	85.7 ± 13.7	90.5 ± 10.7	90.4 ± 12.2	56.5 ± 17.7	81.0 ± 18.5	83.4 ± 18.9	86.4 ± 15.6	90.0 ± 14.4	89.7 ± 13.9
**MODELS**												
3_06_080	51.1 ± 13.9	59.0 ± 15.5	67.3 ± 20.9	73.9 ± 22.3	75.9 ± 24.5	82.8 ± 21.1	62.9 ± 11.8	64.4 ± 20.2	71.3 ± 22.5	73.9 ± 25.9	81.0 ± 22.3	83.8 ± 23.6
3_06_100	51.8 ± 11.6	59.2 ± 16.1	65.6 ± 21.0	74.6 ± 22.9	79.1 ± 23.0	84.7 ± 20.4	62.5 ± 11.1	65.1 ± 21.4	76.1 ± 21.4	81.2 ± 23.4	84.7 ± 21.5	86.4 ± 22.1
3_08_080	50.1 ± 11.7	56.0 ± 16.0	62.0 ± 17.9	68.5 ± 20.7	72.3 ± 23.8	81.5 ± 20.9	62.8 ± 11.4	66.7 ± 20.4	75.6 ± 22.4	77.6 ± 25.6	83.7 ± 22.1	87.0 ± 21.3
3_08_100	50.0 ± 11.5	57.9 ± 14.9	66.7 ± 21.1	73.8 ± 22.2	77.2 ± 23.9	86.5 ± 19.8	61.9 ± 12.3	61.6 ± 21.2	73.8 ± 24.0	79.9 ± 22.7	82.8 ± 23.3	87.2 ± 20.7
3_10_080	51.3 ± 11.9	54.5 ± 16.8	66.9 ± 20.6	71.8 ± 22.4	75.9 ± 24.2	82.7 ± 22.5	62.6 ± 11.7	63.6 ± 19.4	70.3 ± 22.7	75.6 ± 23.6	81.1 ± 21.6	83.6 ± 22.9
3_10_100	51.6 ± 12.5	60.6 ± 17.1	66.3 ± 21.0	73.5 ± 22.8	74.1 ± 25.4	83.8 ± 20.6	63.0 ± 11.4	65.3 ± 21.1	74.7 ± 23.1	79.8 ± 23.8	84.2 ± 22.7	87.7 ± 21.1
4_06_080	50.5 ± 12.3	58.5 ± 16.0	65.6 ± 20.3	71.7 ± 20.8	72.2 ± 24.7	81.6 ± 20.9	61.6 ± 12.2	64.9 ± 20.9	73.5 ± 24.1	76.7 ± 24.4	82.6 ± 22.6	83.7 ± 23.9
4_06_100	52.3 ± 11.8	58.3 ± 15.4	65.5 ± 20.9	71.4 ± 21.8	74.5 ± 23.4	81.9 ± 21.1	62.2 ± 11.8	63.9 ± 20.9	74.0 ± 22.4	76.0 ± 25.5	84.2 ± 21.6	85.7 ± 21.9
4_08_080	50.3 ± 12.6	56.2 ± 14.0	61.0 ± 17.7	70.9 ± 21.5	72.9 ± 24.4	78.4 ± 23.4	64.0 ± 11.1	63.1 ± 21.6	75.3 ± 22.8	77.7 ± 25.1	84.3 ± 20.7	85.0 ± 23.3
4_08_100	51.1 ± 12.1	57.8 ± 15.1	63.3 ± 20.0	68.7 ± 20.8	70.1 ± 24.5	78.7 ± 23.4	62.8 ± 12.7	66.2 ± 20.5	74.8 ± 23.1	77.6 ± 25.2	83.3 ± 21.9	85.9 ± 22.0
4_10_080	49.5 ± 11.3	58.9 ± 15.5	63.0 ± 20.1	69.9 ± 21.1	70.2 ± 25.6	80.0 ± 21.8	63.3 ± 11.3	61.3 ± 20.1	70.0 ± 23.7	73.2 ± 24.7	81.7 ± 21.8	81.9 ± 24.8
4_10_100	51.7 ± 12.5	56.8 ± 16.1	64.5 ± 16.9	68.3 ± 22.2	71.9 ± 24.3	81.3 ± 22.4	63.3 ± 11.7	64.2 ± 20.7	72.6 ± 22.7	79.0 ± 23.2	83.5 ± 22.4	86.3 ± 22.0
5_06_080	51.3 ± 12.2	56.5 ± 14.3	61.0 ± 16.2	66.8 ± 20.1	69.1 ± 23.3	78.8 ± 21.9	61.4 ± 11.7	64.1 ± 20.5	72.8 ± 22.9	74.9 ± 25.0	83.2 ± 21.9	85.1 ± 22.8
5_06_100	53.0 ± 11.6	58.5 ± 16.5	61.3 ± 18.8	65.1 ± 21.2	65.9 ± 23.1	76.2 ± 20.7	59.8 ± 11.9	64.1 ± 19.7	72.0 ± 23.0	74.7 ± 24.3	81.3 ± 21.6	85.9 ± 21.2
5_08_080	50.2 ± 11.2	55.3 ± 16.2	58.6 ± 16.0	64.3 ± 19.5	65.3 ± 22.6	72.6 ± 22.2	58.8 ± 12.2	62.6 ± 19.6	72.7 ± 21.3	75.6 ± 23.1	82.6 ± 21.9	86.6 ± 20.9
5_08_100	49.1 ± 12.0	55.0 ± 14.1	60.7 ± 18.2	67.4 ± 20.2	67.0 ± 23.4	74.1 ± 22.6	61.2 ± 12.0	63.8 ± 21.2	73.1 ± 22.4	73.5 ± 24.1	81.5 ± 21.8	83.4 ± 23.3
5_10_080	50.3 ± 12.5	56.4 ± 14.8	60.8 ± 18.9	68.1 ± 19.7	69.3 ± 23.6	76.5 ± 22.7	62.3 ± 13.4	65.5 ± 21.9	74.9 ± 23.0	76.4 ± 25.7	84.5 ± 20.3	85.5 ± 22.0
5_10_100	50.2 ± 13.1	56.2 ± 14.9	59.4 ± 16.4	64.7 ± 19.2	65.9 ± 23.0	76.0 ± 22.5	62.5 ± 12.0	63.3 ± 21.9	73.6 ± 23.0	79.3 ± 24.3	84.3 ± 21.7	85.8 ± 22.2
**MODELS OVERALL**												
MEAN	50.8	57.3	63.3	69.6	71.6	79.9	62.2	64.1	73.4	76.8	83.0	85.4
MIN	49.1	54.5	58.6	64.3	65.3	72.6	58.8	61.3	70.0	73.2	81.0	81.9
MAX	53.0	60.6	67.3	74.6	79.1	86.5	64.0	66.7	76.1	81.2	84.7	87.7

### Modeled learning curves

**Figure 5:**
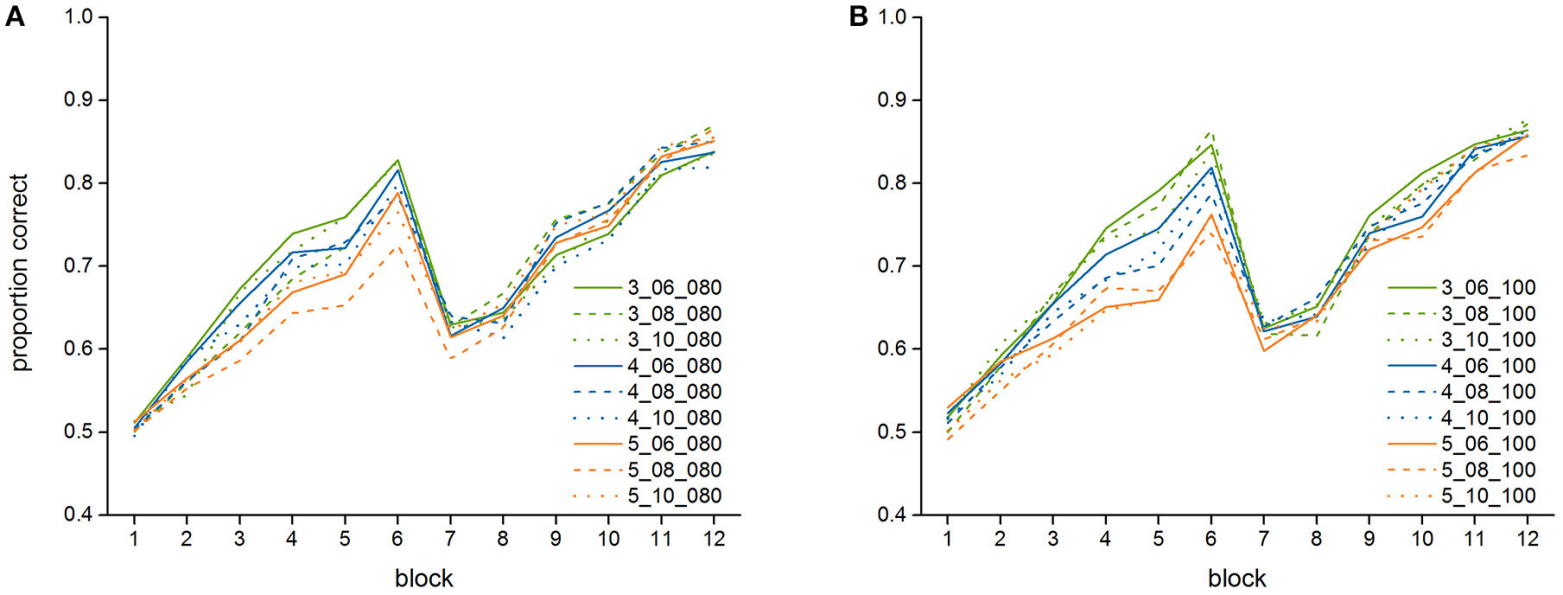
Average performance of the 18 versions of the model in the 12 blocks of the experiment, **(A)** models with a declarative-finst-span of 80 s, **(B)** models with a declarative-finst-span of 100 s.

Figure 4 further shows the means and standard deviations of the proportion of correct responses of the best (3_06_100) and worst fitting (5_10_100) model (see below, Section Model Fit). In addition, Table 2 lists the model performance means and standard deviations for each of the twelve blocks for all 18 models, and Figure 5 shows the learning curves of all 18 models.

Both the best and the worst fitting model (as do all others) capture the overall shape of the learning curve found in the data. They both show an increase in the learning rate in the first six blocks. Similarly, all models show a drop in performance in the seventh block, which is followed by another increase in performance. Even in the best fitting 3_06_100 model, however, the proportion of correct responses is underestimated by the model, especially in the first blocks. Also, the participants show a more severe setback after the switch but then recover faster, while the model takes longer until its performance increases again. Nevertheless, for the best fitting model, the modeled data are always within the range of the standard deviation of the empirical data.

As Table 2 shows, each of the models shows a large degree of variance across its 160 runs. The standard deviation averaged across all 12 blocks ranges from 18.9 to 20.4%, depending on the model's parameter settings. For the best-fitting model, the standard deviation in the individual blocks ranges from 11.6 to 23.4% and is significantly larger than the standard deviation found in the empirical data, except for the first two blocks of the experiment and the first two blocks after the switch (for all blocks except Block 1, 2, 7, and 8: all *F*s > 6.79, all *p*s < 0.010). This high variation of the individual model runs indicates that the same underlying rule-set with the same parameter settings can still result in very different learning curves, depending on which exact strategies are chosen at each point when a new strategy is selected (e.g., initial strategy, alteration of one-feature strategy, alteration of two-feature strategy). Furthermore, similarly to the non-learners among the participants described above (see Section Empirical Learning Curves), not every model run was successful, resulting (for the best fitting model) in a performance below 85% in 35.6% of the runs for Block 6 and in 30.0% of the runs for Block 12.

### Model fit

The average correlation of the model and the empirical data is 0.754. Between 43.9% and 67.1% of the variance in the data is explained by the different models. The average standard deviation of the unexplained variance is 0.136. All *r, r*^2^, and RMSE values for the 18 model versions are presented in Table 3.

**Table 3:** Values of *r, r*^2^, and RMSE of the 18 versions of the model.

	***r***	***r*^2^**	**RMSE**
3_06_080	0.812	0.659	0.124
3_06_100	0.820	0.672	0.109
3_08_080	0.745	0.555	0.134
3_08_100	0.803	0.645	0.119
3_10_080	0.785	0.616	0.132
3_10_100	0.798	0.636	0.114
4_06_080	0.805	0.649	0.128
4_06_100	0.794	0.631	0.124
4_08_080	0.726	0.527	0.138
4_08_100	0.743	0.552	0.135
4_10_080	0.745	0.555	0.146
4_10_100	0.741	0.549	0.133
5_06_080	0.733	0.537	0.146
5_06_100	0.722	0.521	0.152
5_08_080	0.697	0.485	0.164
5_08_100	0.718	0.516	0.158
5_10_080	0.721	0.520	0.144
5_10_100	0.663	0.439	0.156
**OVERALL**			
MEAN	0.754	0.570	0.136
MIN	0.663	0.439	0.109
MAX	0.820	0.671	0.164

As Table 3 and Figure 5 show, the model shows relative robustness to the influence of varying parameter settings. For the first count, a lower value is somewhat better for the fit—there is a stronger increase in the first part of the experiment (until Block 6) for a lower than for a higher first count value. For the second count, a lower value results in a better fit as well. The influence of the declarative-finst-span parameter on the fit-indices is very small, resulting in a slightly better fit either for a declarative-finst-span of 80 s or of 100 s, depending on the settings of first and second count.

The best fit in terms of correlation was achieved for the model with the declarative-finst-span value set to 100 (i.e., the model was able to remember if it had already used a previous strategy for 100 s), a first count of three (i.e., a one-feature strategy needed to be successful at least three times to be considered as “often successful”) and a second count of six (i.e., a two-feature strategy needed to be successful at least six times to be considered as “often successful”). The worst fit was observed for the model with the declarative-finst-span value set to 100, a first count of five and a second count of ten.

The RMSE varies from a minimum of 0.106 (3_06_100) to a maximum of 0.164 (5_08_100). Thus, the model with a first count of three, a second count of six and a declarative-finst-span set to 100 performs best, both in terms of correlation (*r*) and absolute prediction (RMSE).

### Summary

In general, the models predict the data well. The modeled learning curves resemble the form of the average empirical learning curve, with an increase in the first half of the experiment, a short decrease at the beginning of the second half, followed by another increase in performance. The correlation indices of the best fitting model show a good fit, with 67.2% of the variance of the data being explained by the model with a declarative-finst span of 100 s, a first count threshold of three and second count threshold of six. Note that this is also the model with the closest absolute fit (RSME is 0.109).

However, in absolute percentages of correct responses, all of the models perform below the participants in all blocks (except Block 7). Also, the models show greater overall variance than the empirical data. Furthermore, the models are initially less affected by the switch in strategies but take longer to “recover” from the switch in strategies.

In summary, the model replicates the average learning curves and large parts of the variance. It does so with a limited set of rules and the given exemplars, covering learning and relearning processes which take place in dynamic environments. Moreover, we found differences in model fit depending on the exact specification of the parameters, with the best fit if the model remembers previously employed strategies for 100 s, marks a one-feature strategy as “often successful” after three successful uses and a two-feature after six successful uses. However, all of the 18 different parameter settings we tested resembled the main course of the empirical data, thereby indicating that the mechanisms of the model are robust to parameter variations.

## Discussion

The discussion covers three main chapters. First, the fit of the model is discussed and suggestions for possible improvements are given. Second, the broader implications of our approach are elaborated. Finally, future work is outlined.

### Discussion of the modeling approach

Our modeling account covers relevant behavioral data of a dynamic decision-making task in which category learning is required. To solve the task, two features have to be combined, and the relevant feature combination needs to be learned by trial and error using feedback. The model uses feedback from the environment to find correct categories and to enable a switch in the assignment of response buttons to the target and non-target categories. Metacognition is built into the model via processes that govern under what conditions strategic changes, such as transitions from one-feature to two-feature strategies, occur.

Overall, the fit indices indicate that this model solves the task in a similar way as participants do. This includes successful initial learning as well as the successful learning of the reversal of category assignment. Moreover, the observation was made that not all participants are able to solve the task, and the same is observed in the behavior of the modeling approach. Thus, the model is able to generate output data that, on a phenomenological level, resemble those of subjects performing a dynamic decision-making task that includes complex rule learning and reversal processes. Although the overall learning trends found in the data can be replicated well with the general rules implemented in our model, there are two limitations: The variance of the model is larger than that of the participants, and the overall performance of the model is lower than the performance of the participants.

It is likely that the participants have a different and perhaps more specific set of rules than the model. For example, the participants were told which of the two keys to press for the target sound. However, it is unclear if they used this knowledge to solve the task. To keep the model simple, it was not given this extra information, so there was no meaning assigned to the buttons. This is one possibility to explain the model's lower performance, especially in the first block. Another example for more task specific rules used by the participants compared to the model is that the four different features of the stimuli may not be equally salient to the subjects, which may have led to a higher performance compared to the model. For example, it is conceivable that the target-feature direction of frequency modulation (up vs. down) was chosen earlier in the experiment than the non-target feature frequency range, while the model treated all features equally to keep the model as simple as possible. Finally, after the change of the button press rule, some participants might have followed a rule which states to press the opposite key if a strategy was correct for many times and then suddenly is not, instead of trying out a different one- or two-feature strategy, whereas the model went the latter way.

Adding such additional rules and premises to the model would possibly reduce the discrepancy between the performance of the model and the behavioral data. However, the aim of this paper was to develop a modeling approach that incorporates general processes important for all kinds of dynamic decision-making. This implies using only assumptions that are absolutely essential (meta-cognition, switching from one-feature to two-feature strategies, learning via feedback) and keeping the model as simple as possible in other regards. As a consequence, adding extra rules would not produce a better general model of dynamic decision-making, but would only lead to a better fit of the model for a specific experiment while making it prone to overfitting. As mentioned earlier, good descriptive models capture the behavioral data as closely as possible and therefore always aim at maximizing the fit to the data they describe. Good predictive models, on the other hand, should be generalizable to also predict behavior in different, but structurally similar situations and not just for one specific situation with one set of subjects. In our view, this constitutes a more desirable quest with more potential to understand the underlying processes of human dynamic decision-making. This is supported by Gigerenzer and Brighton (2009), who argue that models that focus on the core aspects of decision-making, e.g., considering only few aspects, are closer to how humans make decisions. They also argue that such simplified assumptions make decisions more efficient and also more effective (Gigerenzer and Brighton, 2009).

As stated earlier, one way to model dynamic decision-making in ACT-R using only few assumptions is instance based learning (IBL). This approach uses situation-outcome pairs and subsymbolic strengthening mechanisms for learning. However, IBL is insufficient to model tasks which involve switches in the environment (Fum and Stocco, 2003). Such tasks require adding explicit switching rules. Besides these rules, our task needed mechanisms that control when to switch from simple one-feature strategies to more complex strategies. Since meta-cognitive reflections are not part of IBL, we used a mixed modeling approach which incorporates explicit rules and metacognitive reflection. IBL is insofar part of our approach as the strategies are encoded as situation-outcome pairs and subsymbolic strengthening mechanisms of ACT-R are utilized.

To evaluate if our modeling approach of strategy formation and rule switching is in line with how participants perform in such tasks, data reflecting learning success need to be considered. Such data are the learning curves reported in this paper. We believe that an IBL model alone cannot produce the strong increase in performance after the environmental change in the empirical data.

For a further understanding of complex decision-making, other behavioral data, such as reaction times, could also be modeled. However, not all processes that probably have an impact on reaction time are part of our general modeling approach. This is especially the case for modeling detailed aspects of auditory encoding with ACT-R; for example, the precise encoding of the auditory events can be expected to comprise a different gain in reaction time for short compared with longer tones. However, our modeling approach is expandable, allowing the incorporation of other cognitive processes such as more specific auditory encoding or attention. This extensibility is one of the strengths of cognitive architectures and is particularly relevant for naturalistic decision-making, where many additional processes eventually need to be considered.

### Scope of the model

A formal model was built with ACT-R, it specifies the assumptions of dynamic decision-making in category learning. This model was tested on empirical data and showed similar learning behavior. Assumptions about how dynamic decisions in category learning occur, e.g., by learning from feedback and switching from simple to more complex strategies, and metacognitive mechanisms were modeled together. ACT-R aims at modeling cognition as a whole, thus addressing different cognitive processes simultaneously, an important aspect for modeling realistic cognitive tasks. Moreover, the model is flexible. Thus, the model chooses from the available strategies according to previous experience and random influences.

Our modeling approach is simple in the sense that it comprises only few plausible assumptions, does not rely on extra parameters and is nevertheless flexible enough to cope with dynamically changing environments.

To test the predictive power of the model, it needs to be further tested and compared to new empirical data that are obtained using slightly different task settings. Our aim was to develop a first model of dynamic decision-making in category learning. Thus, relevant cognitive processes that occur between stimulus presentation and the actual choice response are included in the model. Furthermore, we wanted to show how a series of decisions emerge in the pursuit of an ultimate goal. Thus, as a first step we needed a decision task that shows characteristics similar to natural dynamic settings. Such aspects include complex multi-feature stimuli, feedback from the environment, and changing conditions. Since explicit hints on category membership are usually not present in non-experimental situations, it is furthermore reasonable to use a task without explicit instructions regarding which features (or stimuli) attention should be focused on. The downside of using unspecific instructions as done in our study is that from the behavioral data, it will remain unclear how exactly individual participants process such a task, since aspects such as which exact rules are followed or which features are considered at the beginning of a task, are uncertain.

As a next step we aim at modeling and predicting the dynamic decision-making course of individual participants. In general, a big advantage of cognitive modeling approaches is that they can predict ongoing cognitive processes at any point in time. To evaluate the validity of such predictions, different approaches can be followed.

One approach to constructing models in accordance with the cognitive processes of participants is the train-to-constrain paradigm (Dimov et al., 2013). This paradigm requires instructing participants in a detailed step-by-step procedure on how to apply specific strategies in decision tasks. This approach gives the modeler insight into the strategies that participants are using at a given time point. This again can be used to constrain ACT-R models in the implementation of these strategies. In future studies, we plan to adopt this paradigm by (a) instructing the participants and (b) adjusting our model accordingly. To ensure that the train-to-constrain paradigm was successfully implemented, self-reports of the participants should be used.

Another approach is to conduct interviews while the participant is performing the task. To confirm the model's predictions about the prospective behavior of participants, subjects of future empirical studies should thus be asked about their decisions during the course of the experiment. The first few participant decisions can be expected to be strongly influenced by random aspects (e.g., which feature is attended to first), but after some trials, the modeling approach should be able to predict the next steps of the participants. Thus, it should allow precise predictions of the subsequent cognitive processes. To make such predictions, a revised model would need to use the first couple of trials as information about the strategy an individual participant initially follows.

In a further step, the exact cognitive processes proposed by the model should be tested on an individual level on more fine grain data (e.g., fMRI) and then be readjusted accordingly. Currently, different methods to map cognitive models to finer grain data such as fMRI or EEG data have been proposed (Borst and Anderson, 2015; Borst et al., 2015; Prezenski and Russwinkel, 2016a). These methods are currently investigated and have been applied for basic research questions. Nevertheless, mapping cognitive models to neuronal data is a challenge. More research is needed especially for applied tasks. To supplement neuronal data, additional behavioral data, such as button press dynamics (e.g., intensity of button press), can be added as an immediate measurement of how certain an individual participant is about a decision (Kohrs et al., 2014).

Besides using cognitive models to predict individual behavior, we aim to develop more general cognitive mechanisms to model learning, relearning and metacognition that are valid in a broad range of situations. To test the applicability of our modeling approach in a broader context and different situations, variations of the experiment should be tested with different tasks and materials. For example, the model proposed here should be able to predict data from categorization experiments using visual stimuli such as different types of lamps (Zeller and Schmid, 2016) with some modifications to the sensory processing of our model. Furthermore, the model should be capable of predicting data from different types of categorization tasks, for example a task using a different number of categorization features, more switches or different sequences. Such a task would be a predictive challenge for our model; if it succeeds, it can be considered as a predictive model.

The developed general mechanisms can also be used in sensemaking tasks. Such tasks require “an active process to construct a meaningful and functional representation of some aspects of the world” (Lebiere et al., 2013, p. 1). Sensemaking is an act of finding and interpreting relevant facts amongst the sea of incoming information, including hypothesis updating. Performance in our task comes close to how people make sense in the real world because it involves a large number of different stimuli, each carrying different specifications of various features. Thus, “making sense of the stimuli” requires the participants to validate each stimulus in a categorical manner and use the extracted stimulus category in combination with the selected button-press and the feedback that follows as information for future decisions.

To conclude, such a cognitive model which includes general mechanism for learning, relearning and metacognition can prove extremely useful for predicting individual behavior in a broad range of tasks. However, uncertainty remains regarding whether this captures the actual processes of human cognition. This is not only due to the fact that human behavior is subject to manifold random influences, but also to the limitation that a model always corresponds to a reduced representation of reality. The modeler decides which aspects of reality are characterized in the model. Marewski and Mehlhorn (2011) tested different modeling approaches for the same decision-making task. While they found that their models differed in terms of how well they predicted the data, they ultimately could not show that the best fitting model definitely resembles the cognitive processes of humans. To our knowledge, no scientific method is ever able to answer how human cognition definitely works. In general, models can only be compared in terms of their predictive quality (e.g., explained variance, number of free parameters, generalizability). Which model ultimately corresponds to human reality, on the other hand, cannot be ascertained.

### Outlook

One reason for modeling in cognitive architectures is to implement cognitive mechanisms in support systems for complex scenarios. Such support systems mainly use machine learning algorithms. Unfortunately, those algorithms depend on many trials to learn from before they succeed in categorization or in learning in general. Cognitive architecture inspired approaches, on the other hand, can also learn from few samples. In addition, approaches that rely on cognitive architectures are informed models that provide information about the processes involved and the reasons that lead to success and failure.

Cognitive models can be applied to a variety of real-world tasks, for example to predict usability in smartphone interaction (Prezenski and Russwinkel, 2014, 2016b), air traffic control (Taatgen, 2001; Smieszek et al., 2015), or driving behavior (Salvucci, 2006). Moreover, cognitive modeling approaches can also be used in microworld scenarios (Halbrügge, 2010; Peebles and Banks, 2010; Reitter, 2010). Not only can microworld scenarios simulate the complexity of the real world, they also have the advantage of being able to control variables. This implies that specific variations can be induced to test the theoretical approach or model in question (as demonstrated in Russwinkel et al., 2011).

Many applied cognitive models are quite specific task models. Our model, in contrast, aims at capturing core mechanisms found in a variety of real world tasks. As a consequence, it has the potential to be applied in many domains. So, our model of dynamic decision-making in a category learning task makes predictions about the cognitive state of humans during such a task. This involves predictions about strategies (e.g., one-feature or two-feature strategies), conceptual understanding (e.g., assumptions about relevant feature combinations) and metacognitive aspects (e.g., information on the success of the decision maker's current assumption), all of which are aspects of cognition in a multitude of tasks and application domains.

Our general modeling approach therefore has the potential to support users in many domains and in the long run could be used to aid decision-making. For this, the decisions of individual users during the course of a task could be compared to the cognitive processes currently active in the model. If for example a user sticks to a one-feature strategy for too long or switches rules in an unsystematic manner, a system could provide the user with a supportive hint. Other than regular assistant systems, such a support system based on our model would simulate the cognitive state of the user. For example, this online support system would be able to predict the influence of reoccurring negative feedback on the user, e.g., leading him to attempt a strategy change. If, however, the negative feedback was caused by an external source such as a technical connection error, opting for the strategy change would result in frustration of the user. The proposed support system would be able to intervene here. Depending on the internal state of the user, the support system would consider what kind of information is most supportive or if giving no information at all is appropriate (e.g., in case of mental overload of the user). As long as no support is needed, systems like this would silently follow the decisions made by a person.

Moreover, if the goal of the user is known, and the decisions made by the user have been followed by the system, it would be possible to predict the user's next decisions and also to evaluate whether those decisions are still reasonable to reach the goal. Many avalanches have been caused by repeated wrong decisions by backcountry skiers stuck in their wrong idea about a situation (Atkins, 2000). A support system that is able to understand when and why a person is making unreasonable decisions in safety critical situations would also be able to present the right information to overcome the misunderstanding. A technical support system for backcountry skiers would need information about current avalanche danger, potential safe routes and other factors. Such information is already provided by smartphone applications that use GPS in combination with weather forecasts and slope-steepness measures. In the future, when this information is made available to a cognitive model-based companion system that predicts the decisions of the users, it could potentially aid backcountry skiers. Cognitive model-based support systems designed in a similar manner could equally well be employed in other safety-critical domains, as well as to assist cyclist, drivers or pilots.

## Author contributions

AB and SW designed the auditory category learning experiment. SW performed human experiments and analyzed the data. SP and NR designed the ACT-R modeling. SP implemented ACT-R modeling and analyzed the data. SP, SW, and AB prepared figures. SP, NR, and AB drafted manuscript. SP, NR, AB, and SW edited, revised and approved the manuscript.

### Conflict of interest statement

The authors declare that the research was conducted in the absence of any commercial or financial relationships that could be construed as a potential conflict of interest.

## Footnotes

^1^http://act-r.psy.cmu.edu/

^2^Please note that the aural module has two buffers. A tone is first encoded in the aural-location buffer and its content can then be accessed using the aural buffer.

^3^Microworlds are computer simulations of specific problems. They are applied to study real-world problem solving in dynamic and highly complex settings.

^4^The experiments were performed inside an MR scanner to study the specific neural correlates of strategy formation which is the subject of another paper.

^5^The declarative-finst-span parameter controls how long a finst (fingers of instantiation) can indicate that a chunk was recently retrieved. The number of items and the time for which an item can be tagged as attended is limited. These attentional markers are based on the work of Zenon Pylyshyn.
